# Study on Nanoindentation Properties of FCC/B2 Nanostructured Films with Superelastic NiTi Interlayers

**DOI:** 10.3390/ma19061161

**Published:** 2026-03-16

**Authors:** Ranran Fang, Yongyi Deng, Weiping Li, Zhonghua Yan, Jiangen Zheng, Nana Pan, Anatoliy Y. Vorobyev, Dongyang Li, Xiang Chen

**Affiliations:** 1 School of Integrated Circuits, Chongqing University of Posts and Telecommunications, Chongqing 400065, China; fangrr@cqupt.edu.cn (R.F.); dongyang@ualberta.ca (D.L.); chenxiang@cqupt.edu.cn (X.C.); 2 Key Laboratory of Big Data Intelligent Computing, Chongqing University of Posts and Telecommunications, Chongqing 400065, China; 3 School of Electronic Science and Engineering, Chongqing University of Posts and Telecommunications, Chongqing 400065, China; 4 Department of Chemical and Materials Engineering, University of Alberta, Edmonton, AB T6G 2H5, Canada

**Keywords:** molecular dynamics, nanostructured films, nanoindentation, shape memory alloy, martensitic transformation

## Abstract

Dual-phase layered microstructures containing alternating regions of soft and hard phases can produce alloys with a unique combination of strength and ductility. In this study, the molecular dynamics (MD) method was utilized to simulate nanoindentation of a Ni/NiTi/Ni nanostructured film (NSF). This film features a unique alternating FCC/B2/FCC microstructure, in which the B2-phase NiTi acts as a superelastic shape memory alloy (SMA). The results indicate that Ni/NiTi/Ni NSF significantly reduces its hardness due to the superelasticity of the B2 phase. The presence of the NiTi interlayer effectively blocks the propagation path of dislocations and stacking faults by transforming the local dislocations transferred from the upper layer into a large-scale coordinated phase transition, significantly reducing local deformation misalignment. As the thickness of the surface film λ increases, the dislocation slip plane propagating horizontally appears in the upper pure Ni layer. The thicker the surface film, the more horizontal slip planes are formed. This study provides new insights into the contact mechanical behavior of nanostructured films based on NiTi shape memory alloys from the perspective of atomic scale.

## 1. Introduction

Nanostructured film (NSF) can combine a variety of materials with different mechanical properties to form a composite material with unique properties. This composite nanostructured film has attracted significant attention in the field of science and technology due to its high strength, good thermal stability, strong damage resistance, wear resistance, and improved radiation resistance [1,2].

There are various types of nanostructured films, such as FCC/FCC multilayers, FCC/HCP multilayers, BCC(B2)/BCC(B2) multilayers, and FCC/BCC(B2) multilayers. To delve into the mechanical properties of these multilayers, numerous scholars have conducted extensive and systematic research. For instance, some researchers have examined the atomic structure, dislocation mechanism, and mechanical properties of FCC/FCC multilayer films in Cu/Ag composites [3]. Furthermore, scholars have thoroughly investigated the role of BCC/BCC multilayers in Fe/W and other alloys, and have summarized the evolution of their microstructure and mechanical properties under radiation [4]. In the case of FCC/BCC multilayer films, Baras F et al. studied the atomic microstructure and spontaneous crystallization of Ni/Al at the initial state of a fixed temperature, demonstrating their connection to nanostructured films [5].

NiTi shape memory alloys have been shown to have excellent tribological properties due to their superelasticity [6] and have been widely used in various engineering projects, such as scrapers, robotic surgical systems, and biomedicine [7]. To investigate the mechanical properties of Ni/NiTi/Ni under the influence of nanostructured film, nanoindentation technology is introduced. Nanoindentation technology is a scientific and technological method used to accurately measure small deformations and local changes in materials, making it significantly applicable to the study of microscopic material behavior. In a study by Janine Pfetzing-Micklich et al., it was confirmed that the crystal anisotropy of NiTi is controlled by the orientation dependence of martensitic phase transition through nanoindentation technology [8]. In another study by Li, they conducted a parametric evaluation of the microdeformation superelastic behavior of NiTi alloy through nanoindentation technology, focusing on controllable indentation parameters [9].

In addition, molecular dynamics (MD) simulation technology has been proven to be an effective method for atomic-scale simulation [10]. Chen et al.’s MD simulation demonstrates that the tribology of NiTi alloy shows a clear temperature correlation due to superelastic martensitic transformation [11,12]. The mechanism of how superelasticity influences its tribological properties is explained based on the atomic-scale contact morphology. MD simulation is also widely utilized in the research of other materials. For instance, Chen et al. employed MD simulation to investigate the microbehavior of GaN crystal with laser assistance [13], while Gao et al. used MD simulation to analyze the microstructure of FeNiCrCoCu high-entropy alloy under tensile response [14].

The study of the microstructure of nanostructured films based on superelastic NiTi layers is a crucial scientific issue in the field of nanomechanics. Existing molecular dynamics simulations have carried out extensive nanoindentation-related research on NiTi-based materials, mainly focusing on the nanoindentation behavior of pure NiTi shape memory alloys [15], the temperature dependence of their tribological properties [11], and the orientation characteristics of martensitic transformation during nanoindentation [16]. However, none of these studies have explored the FCC/B2 alternating Ni/NiTi/Ni three-layer composite nanostructured film system, nor clarified three core deformation mechanisms: the strain shielding effect of the NiTi interlayer, the KS interface-mediated dislocation slip direction switching, and the coupling effect between martensitic transformation and dislocation blockage. Without the NiTi interlayer, pure Ni exhibits typical characteristics of unconstrained dislocation propagation and uniform stress–strain transmission along the thickness direction under nanoindentation, which is used as a comparison benchmark in this study to clarify the regulatory effect and modification mechanism of the NiTi interlayer. In this paper, molecular dynamics (MD) simulation technology is utilized to systematically investigate the nanoindentation behavior of Ni/NiTi/Ni nanostructured films with FCC/B2 nanolayer structure under varying surface layer thicknesses. We reveal the above three core deformation mechanisms on an atomic scale, and systematically analyze the impacts of NiTi B2 superelastic interlayer and nanostructured film on atomic structure, stress, and strain during nanoindentation, which provides new atomic-scale insights into the contact mechanical behavior of NiTi-based composite nanostructured films.

## 2. Model and Simulation

### 2.1. Model

**Figure 1:**
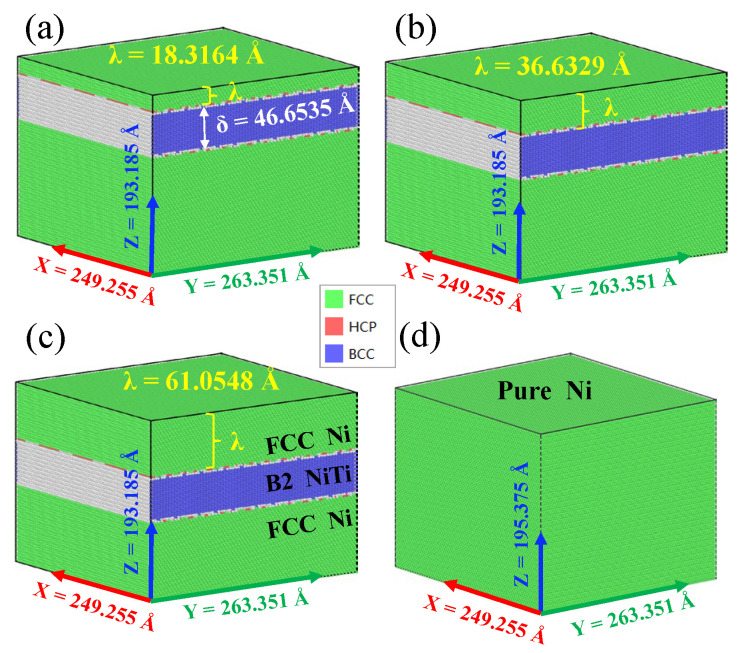
Models of four different Ni/NiTi/Ni nanostructured films: (**a**–**c**) λ = 18.3164 Å, 36.6329 Å, and λ = 61.0548 Å, respectively; (**d**) pure Ni film.

According to research by Daram [17] and Sehitoglu [18], the Ni/NiTi/Ni nanolayer structure consists of an FCC/B2 film. In our simulation model, the FCC/B2 interface follows the classic Kurdjumov–Sachs (KS) orientation relationship [19,20,21]. This KS interface is widely used in heterogeneous structural interfaces [22,23]. Its specific orientation relationship is ${\left[\bar{1}\bar{1}\bar{1}\right]}_{FCC}$//${\left[011\right]}_{BCC}$, ${\left[0\bar{1}1\right]}_{FCC}$//${\left[1\bar{1}1\right]}_{BCC}$. Existing studies have shown that the KS interface is directly related to major deformation mechanisms in a variety of materials [24,25,26]. Figure 1 shows the indentation models of Ni/NiTi/Ni multilayer film with KS interface constructed in this study. The substrate is a single crystal Ni (FCC) structure embedded with a NiTi (B2) superelastic interlayer with a certain thickness (δ = 46.6535 Å), and the NiTi layer is λ from the surface. Single crystal Ni is [011], [21-1], [−11-1] along the X, Y, and Z axes, respectively. The NiTi layer is [−11-1], [21-1], [011] along the X, Y, and Z axes, respectively. Three λ values (18.3164 Å, 36.6329 Å, and 61.0548 Å), corresponding to three, six, and ten times the length along the [−11-1] lattice direction of pure Ni, were examined for their effect on nanoindentation performance. Additionally, a pure nickel model was constructed for comparison. The size and parameter details of all four models are presented in Table 1. After the model is established, periodic boundary conditions and the NPT ensemble are adopted. Energy minimization and relaxation are performed at a temperature of T = 400 K and a pressure of P = 0 GPa until the atomic force is less than 0.01 eV/Å to ensure thermodynamic stability. The time step for the MD simulation is set to 0.001 ps.

### 2.2. Simulation and Analysis

The nanoindentation simulations were performed by Large-scale Atomic/Molecular Massively Parallel Simulator (LAMMPS) [27]. In the present MD simulation, the second nearest neighbour modified embedded-atom method (2NN-MEAM) potential [28], which has been used to study phase transformations [29], compression [30,31], cyclic loading [32], nanoindentation, and nanoscratching [11,15], was selected to describe the interaction between Ni and Ti atoms. The spherical indenter is set to be virtual to eliminate the influence from indenter atoms, which is widely used in nanoindentation MD simulation [33]. In this molecular dynamics simulation, the B2 austenite phase is identified as a BCC structure, and the B19’ martensite phase is recognized as a distorted hexagonal close-packed (HCP) structure for structural analysis, which is a widely accepted simplification in atomistic simulations [34,35,36,37].The force between the indenter and the samples is assumed to be purely repulsive and is expressed as follows:

(1) $$F(r)=\left\{\begin{array}{c} {-K(r-R)}^{2},r<R\\ 0\;\;\;\;\;\;\;\;\;\;\;\;\;\;\;\;\;\;\;,r\geq R \end{array}\right.$$

where K is the stiffness of the indenter, r is the distance from the atom to the indenter center, and *R* is the indenter radius. In this research, K is set to be 10 eV/Å^3^, which is commonly used in the simulations [16,33]. The purely repulsive virtual indenter was adopted and the adhesion effect was neglected in this study, because the interfacial adhesion energy between Ni and the rigid indenter at 400 K is much smaller than the indentation elastic energy, and its influence on the contact mechanical behavior is negligible.

Before conducting the indentation simulation, the Langevin thermostat condition was adopted. In this study, the Langevin thermostat was only applied to the 20 Å atomic shell above the fixed bottom layer of the model, and no thermostat constraint was imposed on the contact zone below the indenter to avoid artificial damping effects on dislocation motion and martensitic transformation dynamics. The 10 Å atoms at the bottom of the model were fixed to prevent the model from moving. In the simulation of the present study, the radius of the indenter was 60 Å, the supplementary indentation rate was 10 m/s. This rate is constrained by the time and spatial scale limitations inherent to MD methods. Consequently, the indentation speed is two to three orders of magnitude higher than that used in physical experiments, although such speeds are commonly employed in MD simulations [38]. Supplementary Figure S1 shows the rate sensitivity verification results of nanoindentation properties for the sample with λ = 36.6329 Å, confirming that the regulation of λ on the nanoindentation properties of the film is an intrinsic structural effect independent of the indentation rate.

The simulation results were analyzed by common neighbor analysis (CNA) [10], dislocation extraction algorithm (DXA) [11], polyhedral template matching (PTM) method [12], and other modules in OVITO visualization software 3.11.3 [9] for atomic structure, dislocation evolution, and phase composition ratio. Additionally, the interaction between dislocations under mechanothermal coupling is closely related to the internal stress distribution. The range of internal stress distribution affects the material’s deformation behavior and mechanical properties. Therefore, the von Mises stress $\sigma_{von}$ distribution was analyzed in this study, and it was used to characterize the state of plastic deformation. It can be observed that the distribution state of internal stress is closely related to the evolution of defects. The formula for calculating $\sigma_{von}$ [13] is as follows:

(2) $$\sigma_{von}=\sqrt{\;\frac{{{(\sigma }_{xx}-\sigma_{yy})}^{2}+{{(\sigma }_{xx}-\sigma_{zz})}^{2}+{{(\sigma }_{zz}-\sigma_{yy})}^{2}}{2}+3{{(\sigma }_{xy}}^{2}+{\sigma_{yz}}^{2}+{\sigma_{xz}}^{2})}$$

where $\sigma_{ij}$(*i*, *j* = *x*, *y*, *z*) represents the stress tensor in the direction *i* parallel to the *j* plane. To verify the reliability and reproducibility of the simulation results, two independent MD simulations were performed for the representative sample with λ = 18.3164 Å. Supplementary Figure S2 confirms that the indentation force–displacement curves and dislocation distribution morphologies of the sample are highly consistent under the same setup, indicating all simulation results in this study have good statistical reproducibility.

## 3. Results and Discussions

When the indenter is pressed into the material, the corresponding load *F* for different penetration depths can be obtained, and the indentation force–displacement (P–h) curve of the penetration process can be obtained. Hardness (H) or contact pressure can be expressed as a function of *F* and the contact area *A_c_* as follows:

(3) $$H=\frac{F}{A_{c}}=\frac{F}{\pi a_{c}^{2}}$$

where, $a_{c}$ is the contact radius. There is no universal equation for calculating the contact radius because it depends heavily on the roughness, size, and shape of the indenter. In this study, we used the idealized approximation proposed by Bushby et al. [39] as follows:

(4) $$a_{c}=\sqrt{R^{2}-{\left(R-h\right)}^{2}}$$

**Figure 2:**
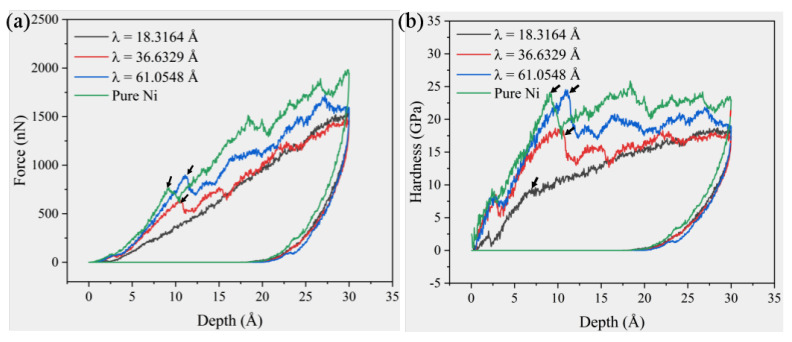
(**a**) Force–displacement curve during the indentation of four samples; (**b**) relationship between hardness evolution (black arrow: the hardness inflection point of the A = 18.3164 A sample with aslowed growth rate).

Since the bottom layer is fixed, the indenter depth *h* is determined by the position of the indenter tip. Figure 2 illustrates the P–h curve of the four models created during the nanoindentation process and the curve depicting the change in hardness during the indentation process. As shown in Figure 2a, as the surface film thickness λ increases, the force during the pressing process also increases, with the pure Ni film exhibiting the highest force. According to the curve, there is no obvious stress drop point (pop-in) during the pressing process of Ni/NiTi/Ni nanostructured film with λ = 18.3164 Å. The pop-in becomes more pronounced as λ increases, and the required force gradually rises, as indicated by the black arrow in Figure 2a. There is little difference between Ni/NiTi/Ni nanostructured films in the unloading phase. The hardness curve in Figure 2b shows that hardness increases gradually during the indentation process, dropping sharply when the pop-in occurs. For pure Ni films, the hardness then stabilizes at a relatively constant level, consistent with the research findings for Al and Fe [40]. In the case of Ni/NiTi/Ni nanostructured film, the stable hardness value increases with λ. At λ = 18.3164 Å, the hardness exhibits a clear inflection point, as shown by the black arrow in Figure 2b. Beyond the inflection point, the hardness increases at a slower rate than in the previous stage, rather than remaining at a stable value.

**Figure 3:**
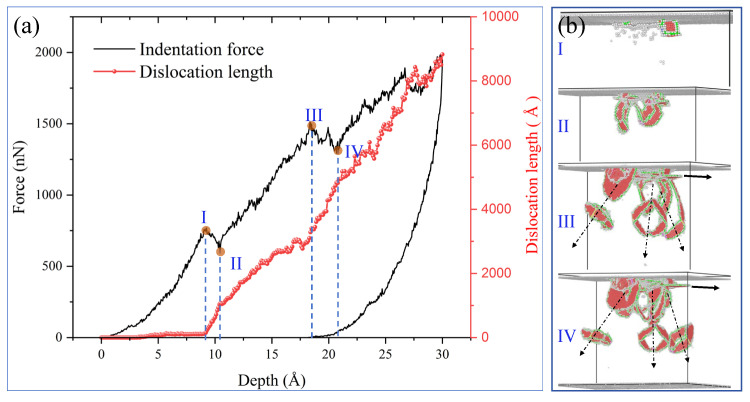
(**a**) Correlation between P–h curves and dislocation evolution during pure Ni indentation; (**b**) snapshots of dislocation and atomic structure corresponding to specific indentation position. Roman numerals I–IV mark key indentation depths. black arrows in (**b**) indicate the formation of three directional radiating dislocation loops inside the sample.

In order to further analyze the microscopic mechanism corresponding to pop-in, this study presents the relationship between the P–h curve and dislocation evolution in the indentation process, as illustrated in Figure 3. As shown in Figure 3a,b, it can be clearly observed that as the head keeps pressing in, the indentation depth increases, and the length of the dislocation gradually increases. Previous studies suggested that the evolution of plastic deformation was closely linked to the pop-in of the indentation curve [18]. As depicted in Figure 3a, the dislocation density within the sample was initially very low and almost negligible before the pop-in. Upon reaching the starting point I of the first pop-in, it is evident from Figure 3b that a noticeable dislocation has formed. As the indentation progresses from point I to II, the dislocation density starts to increase rapidly, and the dislocation structure in Figure 3b transforms from a single dislocation to a pattern of dislocation groups with triple symmetry evolution. Transitioning from point II to III, during the phase of force rise, the rate of dislocation density increase slows down, and by reaching III, a radiating dislocation loop in three directions is formed internally. Moving into the second pop-in from III to IV, the rate of dislocation increment significantly rises. These results indicate that the occurrence of pop-in events is not directly linked to the formation and emission of dislocation loops but is closely associated with the rate of increase in total dislocation density.

**Figure 4:**
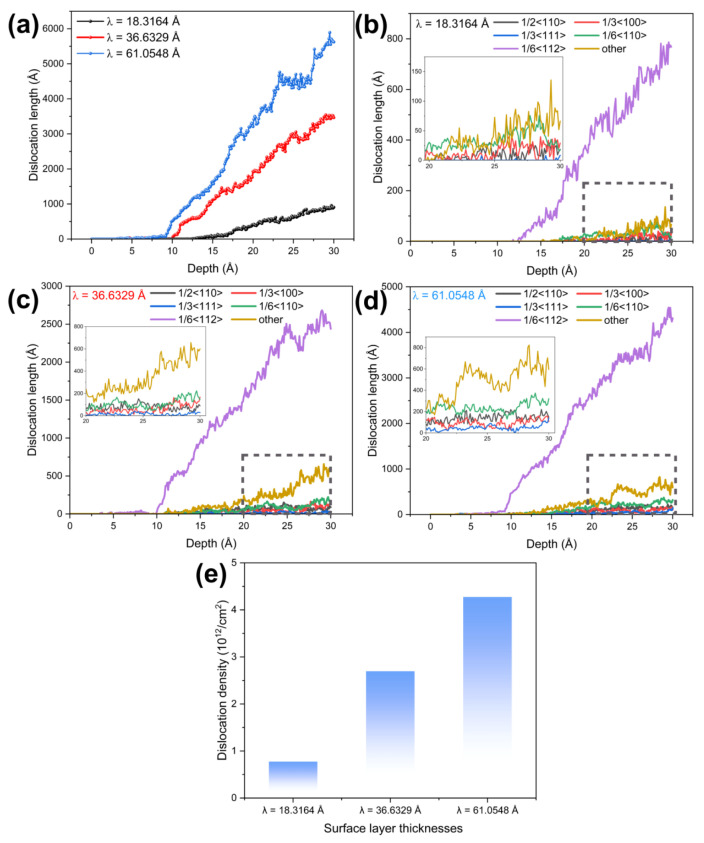
(**a**) Total dislocation length; (**b**–**d**) the evolution of various types of dislocation lengths with indentation depth for films with λ = 18.3164 Å, 36.6329 Å, and 61.0548 Å, respectively; (**e**) dislocation density of the upper Ni layer at an indentation depth of 30 Å (the NiTi interlayer and the lower Ni layer have no dislocations).

**Figure 5:**
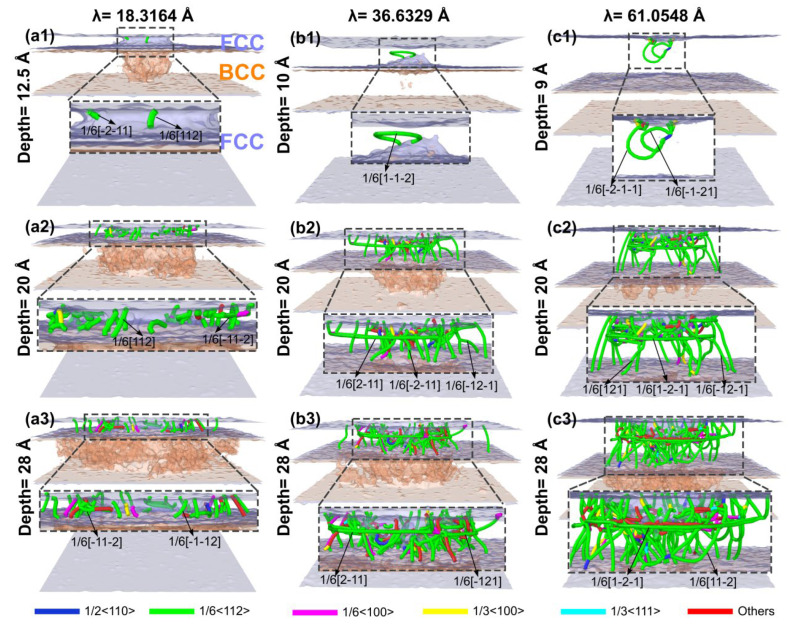
The indentation-induced dislocation configurations and phase structures in Ni/NiTi/Ni films with varying surface layer thicknesses (λ): (**a1**–**a3**) λ = 18.3164 Å; (**b1**–**b3**) λ = 36.6329 Å; (**c1**–**c3**) λ = 61.0548 Å.

**Figure 6:**
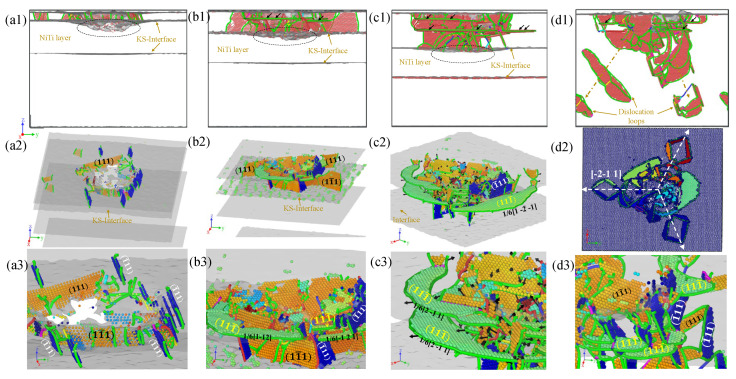
Lateral views of dislocation structures and local dislocation/stacking fault details for four samples. (**a1**–**a3**) λ = 18.3164 Å; (**b1**–**b3**) λ = 36.6329 Å; (**c1**–**c3**) λ = 61.0548 Å; (**d1**–**d3**) pure Ni. Black dashed lines indicate the variation in KS interface concavity with film thickness; black arrows in (**b1**–**d1**) mark the formation and increase of horizontal slip planes in the upper pure Ni layer.

The results show that the total dislocation length in all three types of films increases with greater indentation depth (Figure 4a), but significant differences exist in the critical indentation depth at which dislocation growth initiates markedly and in the subsequent growth rates. Specifically, for the film with λ = 18.3164 Å, the length of 1/6<112>-type dislocations increases rapidly after exceeding a critical depth of 12.5 Å, while other dislocation types show only minor fluctuations. In the film with λ = 36.6329 Å, the pronounced growth of this dislocation type starts earlier, at approximately 10 Å, and maintains a relatively high growth rate throughout the process. The film with λ = 61.0548 Å exhibits the smallest critical initiation depth (about 9 Å) and continues to show sustained high-speed growth even after the depth exceeds 20 Å. This phenomenon is closely related to the stress response characteristics of the NiTi interlayer: a thinner surface layer requires the indenter load to overcome the constraints of the Ni layer and the interactions at the KS interface to trigger the stress-induced martensitic transformation in the NiTi layer, thereby initiating dislocation motion. Consequently, a larger critical indentation depth is required for dislocation activation. Furthermore, the confined space within thinner surface layers is less conducive to the formation of multiple horizontal slip planes (corresponding to the (11-1) slip plane). As the indentation depth continues to increase, a larger λ corresponds to a faster increase in total dislocation length, which is attributed to the expansion of dislocation slip space and the activation of a greater number of slip systems due to the increased surface layer thickness. Further analysis of the evolution of various dislocation types Figure 4b–d reveals that, regardless of the surface layer thickness, 1/6<112>-type dislocations remain the dominant growth type during indentation, with their length growth rate significantly higher than that of other dislocations such as 1/2<110> and 1/3<111>. After increasing the surface layer thickness, the number of “Other”-type dislocations significantly exceeds that of dislocations like 1/2<110> and 1/3<111>, as shown in the locally magnified dashed section of the figure. To further quantify the dislocation distribution characteristics at the critical indentation depth of 30 Å, the dislocation density of each regional layer is calculated and presented in Figure 4e. The results show that dislocations are exclusively concentrated in the upper pure Ni layer, and the dislocation density of the upper Ni layer increases remarkably with the increase of surface layer thickness λ. Notably, no dislocations are detected in the NiTi interlayer and the lower pure Ni layer at this indentation depth, which directly demonstrates that the NiTi interlayer can effectively block the propagation of dislocations from the upper layer to the lower layer, and further verifies the excellent deformation shielding effect of the NiTi interlayer, which is highly consistent with the spatial distribution characteristics of dislocations shown in Figure 5 and Figure 6.

Figure 5 visually elucidates the influence of the NiTi interlayer and the surface layer thickness (λ) on the spatial configuration and type distribution of dislocations in Ni/NiTi/Ni nanostructured thin films. It presents the microscopic dislocation morphology and the spatial distribution features of typical dislocation types for films with λ = 18.3164 Å, 36.6329 Å, and 61.0548 Å during nanoindentation. Combined with DXA analysis results, the slip directions and evolution patterns of various dislocations are further clarified, forming a contrast with the dislocation configuration of the pure Ni film shown in Figure 3. The figure reveals that dislocations are primarily concentrated within the surface FCC layer. While stress-induced atomic misalignment defects are evident in the NiTi interlayer, no distinct dislocation lines form there. Similarly, no significant dislocations are observed in the underlying FCC layer. At their respective critical dislocation initiation depths, all three films exhibit the dominant nucleation of 1/6<112>-type dislocations. For the film with λ = 18.3164 Å (Figure 5(a1)), dislocations display a distinct inclined distribution along crystallographic planes with no indication of horizontal slip. This is attributed to the spatial constraint imposed by the KS interface in thinner surface layers, which promotes easier dislocation nucleation and motion at the interface. In the film with λ = 36.6329 Å (Figure 5(b1)), dislocations are primarily oriented horizontally, extending along the crystal planes, which contrasts with the inclined pattern observed at the smallest λ. The thickening of the surface layer expands the slip space within the upper Ni layer and weakens the constraining effect of the KS interface, thereby shifting the dominant slip direction from inclined to horizontal. For the film with λ = 61.0548 Å (Figure 5(c1)), the horizontal distribution persists, with slip segments appearing more continuous. The thick surface layer provides ample space for horizontal slip, and the delayed initiation of the phase transformation in the NiTi layer further reduces constraints on dislocation direction.

**Figure 7:**
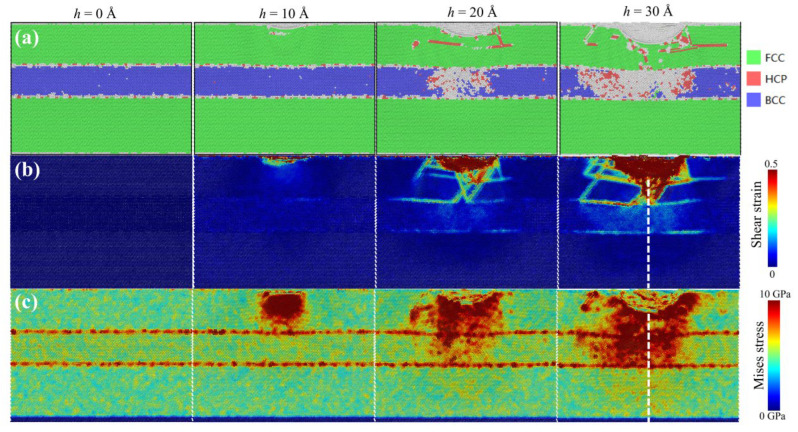
The evolution of (**a**) atomic structure; (**b**) strain and (**c**) Mises stress in Ni/NiTi/Ni nanocomposite films with λ = 61.0548 Å at indenting depth of 0 Å, 10 Å, 20 Å and 30 Å, respectively.

At an indentation depth of 20 Å, 1/6<112>-type dislocations continue to grow rapidly. Figure 5(a2) shows the formation of inclined slip bands traversing the upper Ni layer, while Figure 5(b2,c2) exhibits continuous horizontal slip bands and a networked horizontal slip structure, respectively. The population of secondary dislocation types (1/6<110>, 1/2<110>) increases. The martensitic transformation in the NiTi layer becomes more extensive, with a higher transformation intensity observed in films with smaller λ. This trend corroborates the localized characteristic described in Figure 7, where “strain is completely hindered in the central region of the NiTi layer.”

**Figure 8:**
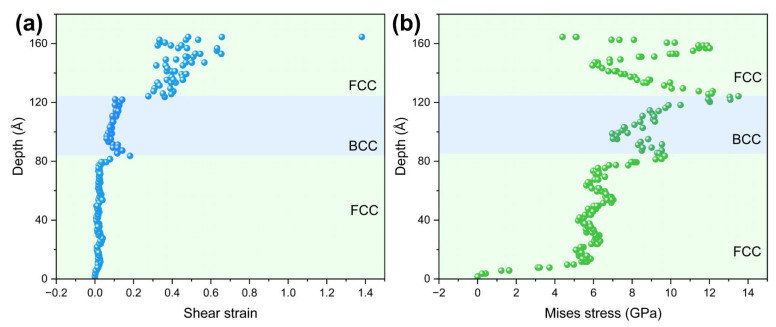
Variation of strain and von Mises stress with depth at the white line position (Figure 7) in Ni/NiTi/Ni nanocomposite films with λ = 61.0548 Å under an indentation depth of 30 Å: (**a**) strain distribution; (**b**) Mises stress distribution.

**Figure 9:**
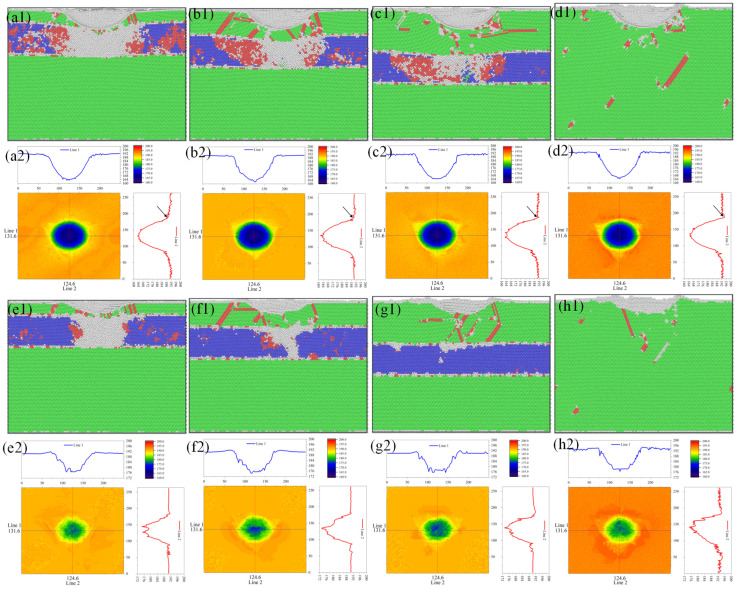
Section snapshot of atomic structure of samples before unloading (**a1**) λ = 18.3164 Å; (**b1**) λ = 36.6329 Å; (**c1**) λ = 61.0548 Å; (**d1**) distribution map of pure Ni and surface morphology (**a2**–**d2**). Sectional snapshot of atomic structure (**e1**–**h1**) and surface topography (**e2**–**h2**) after unloading.

By an indentation depth of 28 Å, the dislocation configuration stabilizes. The length of 1/6<112>-type dislocations reaches its maximum while maintaining their initial directional characteristics. The martensitic transformation in the NiTi layer peaks and remains stable. A smaller λ corresponds to a lower proportion of the B2 phase, leading to earlier limitations on dislocation multiplication, whereas a larger λ promotes the development of a more extensive dislocation network. In summary, the NiTi interlayer effectively converts localized dislocations transmitted from the upper layer into a large-scale, coordinated phase transformation via stress-induced martensitic transformation, thereby blocking dislocation propagation to the underlying layer. The surface layer thickness (λ) not only regulates the available dislocation slip space and the intensity of the phase transformation response but also directly determines the dominant slip direction during the initiation stage, with 1/6<112>-type dislocations consistently playing the leading role. These findings are highly consistent with the core conclusions of the abstract and the evolutionary patterns shown in Figure 6, Figure 8 and Figure 9, clearly revealing the dual dependency of the microscopic deformation mechanisms in Ni/NiTi/Ni nanostructured thin films on both indentation depth and layer thickness.

The dislocation behavior and the evolution of the stacking fault (SF) in four different samples during the nanoindentation process are discussed here. The final compression depth of all four samples was 30 Å. This will help us understand the role of the Ni/NiTi/Ni nanostructured film in the nanoindentation process and the impact of varying surface thicknesses on the samples. Figure 6 illustrates the lateral view of the spatial distribution of dislocation structures with λ values of 18.3164 Å, 36.6329 Å, 61.0548 Å, and pure Ni, respectively, along with local details of the dislocation and stacking fault. It is evident from the figure that for pure Ni, the sample surface constructed is the (−11-1) surface, and the dislocation spreads across the sample space emitting a dislocation loop following a triple symmetric pattern (see Figure 6(d2)). In the case of Ni/NiTi/Ni nanostructured films, the indentation dislocations are concentrated above the NiTi layer, and no stacking faults or dislocations are observed below the indentation process. This indicates that the presence of the NiTi interlayer effectively blocks the dislocation propagation path and stacking faults. More defects are observed at the KS interface of the upper layer. As the film thickness increases, the number of defects and the concavity of the KS interface decrease, as indicated by the black dashed line in the figure. For the surface film with λ = 18.3164 Å, the fault plane mainly inclines towards the (−111) and (111) planes, constrained by the height direction of the NiTi layer, causing the dislocation to propagate in the plane direction. As the thickness of the surface film λ increases, the dislocation slip plane propagating horizontally appears in the upper pure Ni layer. The thicker the surface film, the more horizontal slip planes are formed, as depicted by the black arrow in the Figure 6(b1–d1). The horizontal slip plane corresponds to the (11-1) plane, and the partial dislocations on the slip plane are 1/6[1-2], 1/6[1-2-1], and 1/6[2-1 1].

In order to investigate the impact of Ni/NiTi/Ni nanostructured film on atomic structure and mechanical properties, the atomic structure, strain, and stress distribution of Ni/NiTi/Ni samples with a surface thickness of λ = 61.0548 Å at different positions of the indenter are presented. From Figure 7a, it is evident that the atomic structure changes are not influenced by the NiTi sandwich film in the initial stage of indenter embedding due to shallow indenter penetration. However, with the continuous penetration of the indenter, when the indenter reaches a depth of h = 20 Å, a significant number of structural phase transitions can be observed in the NiTi layer. Furthermore, at a penetration depth of h = 30 Å, as depicted in Figure 7a, more phase transition atoms appear in the middle layer of NiTi. This phenomenon is associated with stress-induced martensitic transition (MT). Comparing with Figure 7c, it is evident that the distribution of phase transitions in the NiTi layer aligns with the stress distribution in the NiTi layer. In Figure 7b, a clearer observation can be made on how the NiTi layer in the Ni/NiTi/Ni nanostructured film weakens the strain propagation of the sample. As the pure Ni in the upper layer comes into direct contact with the indenter, the generated strain is transferred to the NiTi intermediate layer. Through the strain coordination of the NiTi layer, the strain transfer is completely obstructed in the middle of NiTi, resulting in a wide range of atomic strain, while the strain in the lower Ni layer approaches 0, consistent with the findings in Figure 6. In comparison with the local atomic rearrangement of stacking fault formation and dislocation evolution, the phase transition behavior exhibits characteristics of large-scale transient evolution. In this manner, the NiTi intermediate layer transforms the layer dislocations and local dislocations from the upper layer into large-scale coordinated phase transitions, significantly reducing the inconsistency of local deformation. Simultaneously, the strain and stress exhibit regional characteristics, being confined to the areas above the NiTi layer and nearly blocking the transmission of stress and strain from the bottom region of the NiTi layer, thereby achieving a deformation shielding effect.

**Figure 10:**
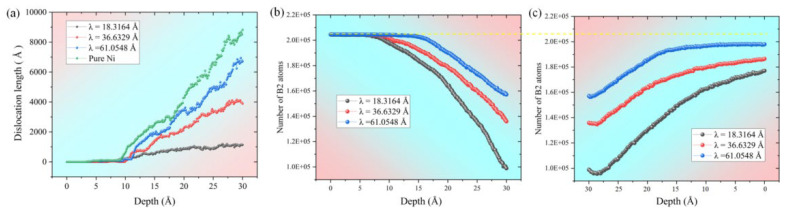
(**a**) Dislocation evolution in pure Ni layer during indentation of four samples; evolution of B2 structure in NiTi layer during loading (**b**) and unloading (**c**).

To further quantitatively characterize the layered attenuation feature of stress and strain in the Ni/NiTi/Ni nanostructured films, the variation of strain and von Mises stress with depth at the white line position (Figure 7) for the sample with λ = 61.0548 Å at 30 Å indentation depth is presented in Figure 10. It can be clearly seen that the stress and strain maintain high values in the upper Ni layer, decrease rapidly when passing through the NiTi interlayer, and drop to nearly zero in the lower Ni layer. This distinct layered attenuation characteristic directly verifies the effective deformation shielding effect of the NiTi interlayer, which is consistent with the previous results that dislocations are confined above the NiTi interlayer and phase transformation is concentrated in the NiTi interlayer. The stress and strain transmission from the upper Ni layer to the lower Ni layer is almost completely blocked by the NiTi interlayer, which is the key reason for the localized deformation of the Ni/NiTi/Ni nanostructured films. Supplementary Figure S3 further presents the depth-dependent evolution of atomic strain and von Mises stress for samples with different surface layer thicknesses, verifying the deformation shielding effect of the NiTi interlayer and the regulatory role of λ in stress–strain distribution.

Figure 9 displays the section diagrams of the atomic structure of the four samples before and after unloading, along with the overhead profile diagram of the surface topography. Through Figure 9, we can investigate whether the outer surface of the sample exhibits any special behavior during the indenter pressing process. Initially, by comparing the section diagrams of the atomic structure of different surface thicknesses before unloading, it is observed that as the surface thickness increases, the atomic proportion of martensitic phase transition in NiTi sandwich gradually decreases, and the phase transition range and the number of amorphous atoms produced also decrease gradually. The surface topography profile reveals that samples with varying surface thicknesses all exhibit triple symmetries on the outer surface after nano pressing, and these symmetries become more pronounced with the increase of λ. A slight variation is noticeable in the section curve after the indenter is pressed in; as λ increases, the curve transitions from a local depression of the pressed part to a local pile-up. This suggests that the presence of the NiTi interlayer can influence the surface morphology of the sample through local deformation coordination.

Then we compare the sectional view of the atomic structure before and after unloading, as well as the top view of the surface morphology. By comparing the sectional diagram of the atomic structure before and after unloading, we can clearly observe that the indentation of the sample changes from smooth and flat before unloading to uneven after unloading, which is also well reflected in the sectional curve of the surface topography distribution diagram. This phenomenon is caused by the superelasticity of the NiTi interlayer of the sample. Before unloading, the indentation remains smooth due to the presence of an indenter. When the indenter is unloaded, the sample will bounce back due to its superelasticity. Moreover, this superelasticity is more clearly reflected in the topography distribution diagram, and we can find that the position height of the indentation after unloading is obviously higher than that before unloading, which is the superelasticity of the sample. In addition, with the increase of λ, the pile-up atoms on the surface after unloading increase, and the most pile-up atoms ard on the pure Ni surface.

According to Figure 10a, it can be observed that the dislocation of the Ni layer increases with the indenter penetration depth. This is due to the continuous activation of the slip system as the indenter penetrates the sample, leading to dislocation glide in the Ni layer and a subsequent increase in dislocation. Furthermore, when compared to the Ni/NiTi/Ni nanostructured film, it is evident that the pure Ni sample exhibits the highest increase in dislocation, while the sample with a surface thickness of λ = 18.3164 Å shows the least increase. This trend is also reflected in the dislocation spatial distribution diagram in Figure 6. The absence of Ni/NiTi/Ni nanostructure in the pure Ni sample allows for more space for dislocation propagation, potentially forming dislocation rings. As the surface thickness decreases, the rate of dislocation increase also decreases. This is attributed to the limited space for dislocation generation and propagation with smaller surface thickness, resulting in earlier hindrance by the sample interlayer and a slower dislocation slip velocity. The graph illustrates that the presence of the NiTi interlayer hinders dislocation propagation by impeding the dislocation slip velocity.

In Figure 10b, it can be observed that the evolution of the B2 structure in samples with different surface thicknesses varies. The B2 structure in samples with smaller surface thicknesses decreases earlier and at a faster rate. This is because the B2 structure is primarily concentrated in the middle layer of NiTi. When the surface thickness of the sample is smaller, the change induced by the indenter will reach the NiTi layer sooner, leading to stress-induced MT, as analyzed in Figure 7. Therefore, the thinner the surface thickness, the earlier the decrease in the B2 structure. Moreover, with a smaller surface thickness, the impact of the indenter-induced change on the Ni/NiTi/Ni nanostructured film is more significant, resulting in more NiTi layer MT and a quicker reduction in the B2 structure, as clearly depicted in Figure 10.

In Figure 10c, it can also be found that the B2 structure has a certain recovery ability during unloading, which is related to the reverse MT after stress reduction, and this recovery ability is strongly correlated with the surface thickness of the sample. The greater the surface thickness of the sample, the better the final recovery effect. This recovery ability is a reflection of NiTi superelasticity, and because the NiTi layer of the sample with a large surface thickness is less affected by the local plasticity of the indenter, fewer amorphous atoms are produced, so the recovery effect is better. However, none of the samples can be restored to the original state, as shown by the yellow dotted line in Figure 10. We can conclude that the superelasticity of the sample has a certain limit, which is also reflected in Figure 6.

## 4. Conclusions

To summarize, molecular dynamics (MD) simulations were conducted to investigate the fundamental deformation behavior of Ni/NiTi/Ni nanostructured films under nanoindentation using a spherical indenter. The study revealed that the P–h curves of pure Ni under nanoindentation are higher than those of Ni/NiTi/Ni nanostructured films. Additionally, the inclusion of a NiTi interlayer decreased the hardness of the film. The presence of the NiTi interlayer effectively blocks the propagation path of dislocations and stacking faults by transforming the local dislocations transferred from the upper layer into a large-scale coordinated phase transition, significantly reducing local deformation misalignment. Strain and stress exhibited localized deformation. A large amount of strain and stress appeared in the upper Ni layer, due to the KS interface, the middle NiTi layer has been significantly reduced, and there is almost no stress and strain in the lower Ni layer. The surface topography profile showed that samples with varying surface thicknesses all displayed triple symmetries on the outer surface after nano pressing. These symmetries became more pronounced with the increase of λ. As λ increased, the curve transitioned from a local depression of the pressed part to a local pile-up, and the rate of dislocation increase also rose.

## Figures and Tables

**Table 1:** Four types of model sizes and their related parameters.

Name	*λ* (Å)	X (Å)	Y (Å)	Z (Å)	*δ* (Å)	Total Number of Atoms
Ni/NiTi/Ni-I	18.3164	249.255	263.351	193.185	46.6535	1,106,496
Ni/NiTi/Ni-II	36.6329	249.255	263.351	193.185	46.6535	1,106,496
Ni/NiTi/Ni-III	61.0548	249.255	263.351	193.185	46.6535	1,106,496
Pure Ni	195.375	249.255	263.351	195.375	0	1,171,200

## Data Availability

The original contributions presented in this study are included in the article/Supplementary Material. Further inquiries can be directed to the corresponding author.

## Supplementary Materials

The following supporting information can be downloaded at: https://www.mdpi.com/article/10.3390/ma19061161/s1, Figure S1: Rate sensitivity of Ni/NiTi/Ni film (λ = 36.6329 Å); Figure S2: MD simulation repeatability of Ni/NiTi/Ni film (λ = 18.3164 Å); Figure S3: Atomic strain/stress distribution and depth variation of Ni/NiTi/Ni films (30 Å indentation depth).
