# Supplementary material for: Study on Nanoindentation Properties of FCC/B2 Nanostructured Films with Superelastic NiTi Interlayers
Source: Materials (Basel). 2026 Mar 16;19(6):1161. doi: 10.3390/ma19061161 (PMC13027521; doi:10.3390/ma19061161)
Supplement: Supplementary file 1 [file materials-19-01161-s001.zip › materials-4171060-supplementary.pdf]

Appendix A. Supplementary Data

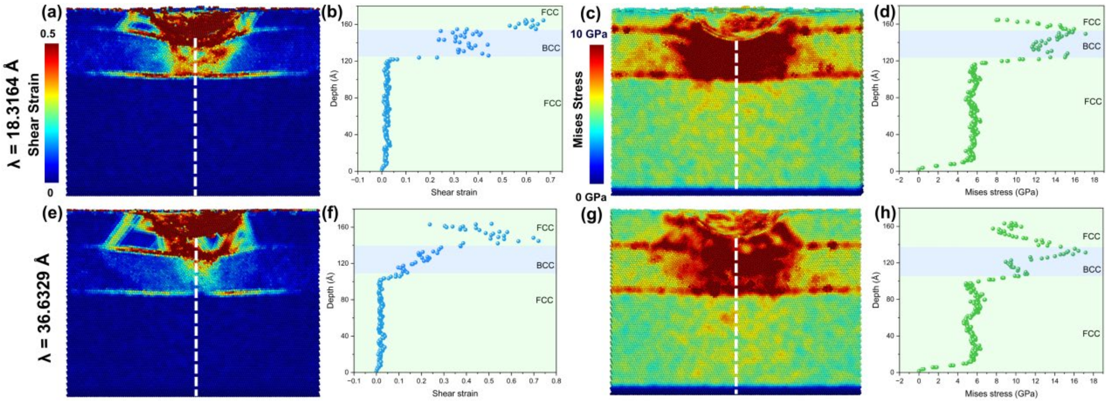

Fig. S1 Atomic strain/stress distribution and their depth-dependent variation curves of Ni/NiTi/Ni nanocomposite films at an indentation depth of 30 Å: (a, b) λ = 18.3164 Å (strain distribution, strain-depth curve); (c, d) λ = 18.3164 Å (stress distribution, stress-depth curve); (e, f) λ = 36.6329 Å (strain distribution, strain-depth curve); (g, h) λ = 36.6329 Å (stress distribution, stress-depth curve)

Fig. S1 clearly reveal the consistent evolution law of stress and strain in the films with different surface layer thicknesses: the atomic strain and Mises stress are highly concentrated in the upper pure Ni layer under the indenter, showing obvious high-value distribution characteristics, and then decrease rapidly when passing through the NiTi interlayer, and the stress and strain values in the lower Ni layer are close to zero, which directly reflects the significant layered attenuation characteristic of stress and strain along the depth direction of the films. Meanwhile, the difference in surface layer thickness λ also leads to the difference in the stress-strain distribution and attenuation process: compared with the sample with λ= 18.3164 Å, the high-value range of stress and strain in the upper Ni layer of the sample with λ= 36.6329 Å is wider, the attenuation rate in the NiTi interlayer is relatively slower, and the start position of attenuation is more downward. These results further supplement and verify the excellent deformation shielding effect of the NiTi interlayer, which can effectively

block the transmission of stress and strain from the upper layer to the lower layer, and also confirm the regulatory effect of surface layer thickness on the internal stress-strain transmission behavior and distribution characteristics of Ni/NiTi/Ni nanostructured films, which is consistent with the core conclusions of the main text on the stress-strain regulation mechanism of the NiTi interlayer and surface layer thickness.

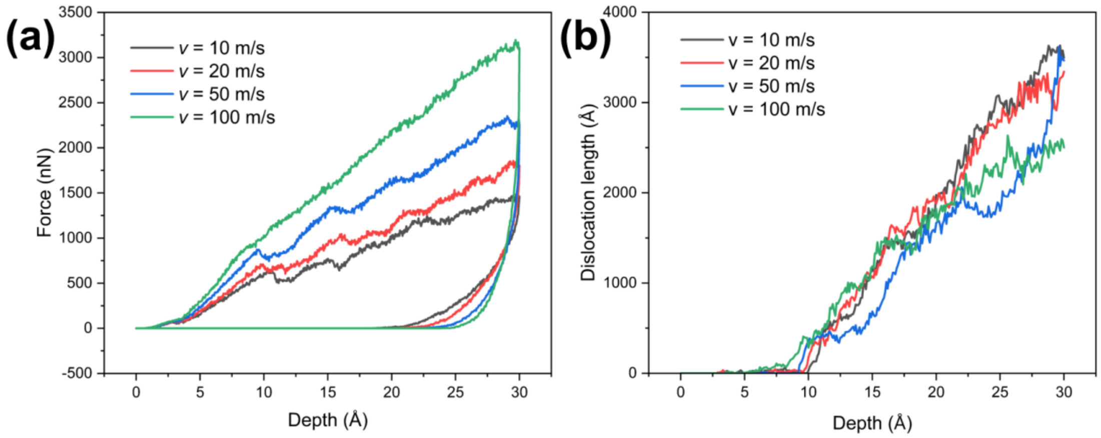

Fig. S2 Rate sensitivity verification of nanoindentation properties for Ni/NiTi/Ni nanostructured film with λ = 36.6329 Å: (a) Comparison of indentation force-displacement (P-h) curves under four indentation rates (10 m/s, 20 m/s, 50 m/s, 100 m/s); (b) Comparison of total dislocation length evolution with indentation depth under four rates (no dislocations in the NiTi interlayer and lower Ni layer for all rates).

The indentation rate adopted in the original MD simulation was 10 m/s (rather than 20 m/s as described in the main text), which is a conventional rate constrained by the spatiotemporal scale of MD simulations [34]. Fig. S2(a)shows the comparison of P-h curves within the indentation depth range of 0~30 Å, where all four curves present a highly consistent monotonous rising trend without obvious stress drop or pop-in point offset, and the indentation force only increases slightly with the rise of rate at the same depth, with the curve spacing remaining stable throughout the process. Fig. S2(b) displays the evolution comparison of total dislocation length in the same

depth range, all four curves follow the consistent rule of "slow increase at 0~10 Å, rapid increase at depth greater than 10 Å" with the increase of indentation depth. The total dislocation length increases slightly with the rise of rate at the same depth, and dislocations are only concentrated in the upper Ni layer at all rates, with no dislocations detected in the NiTi interlayer and lower Ni layer during the whole process. In general, within the rate range of 10~100 m/s, the evolution of P-h curves and total dislocation length of the film show highly consistent trends, which confirms that the regulation of surface layer thickness λ on the nanoindentation properties of the film is an intrinsic structural effect of the material rather than an artifact caused by indentation rate, and also verifies that the dislocation shielding effect of the NiTi interlayer is rate-independent.

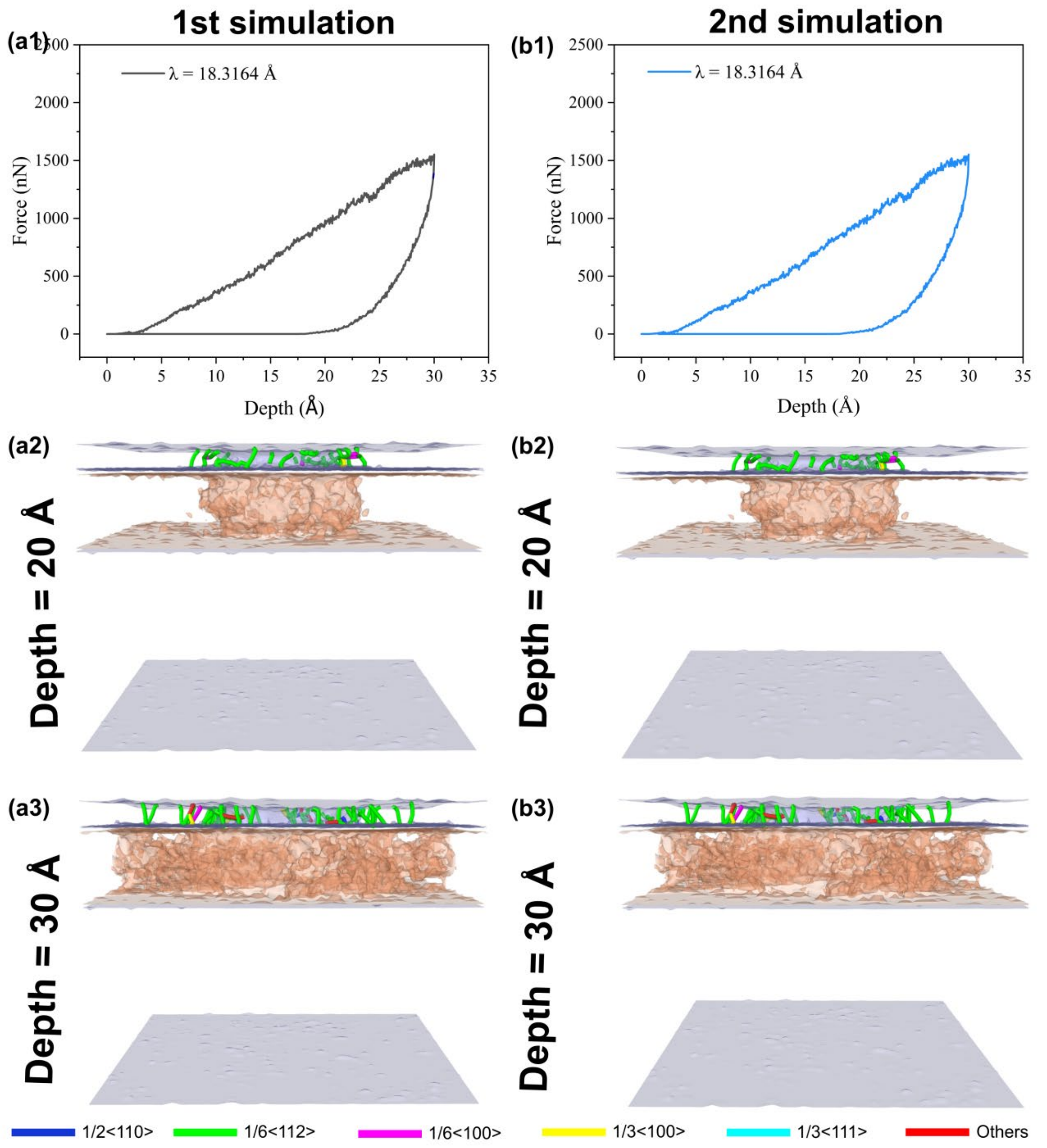

Fig. S3 Repeatability verification of MD simulation results for Ni/NiTi/Ni nanostructured film with λ = 18.3164 Å at an indentation rate of 10 m/s. (a1, b1) Comparison of indentation force-displacement (P-h) curves from two independent runs under identical initial conditions; (a2, b2, a3, b3) Comparison of dislocation distribution morphologies at indentation depths of 20 Å and 30 Å.

To confirm the repeatability of our simulation results, we performed two independent MD simulations for the representative case (λ = 18.3164 Å, 10 m/s) under identical initial conditions and simulation parameters. As shown in Fig. S3, the P–h curves (a1, b1) and dislocation distribution morphologies (a2–a3, b2–b3) from the two runs are virtually identical. This perfect consistency demonstrates that the

nanoindentation response and deformation mechanism of the Ni/NiTi/Ni films are deterministic and highly reproducible under the given simulation setup, rather than random or occasional results. Therefore, all conclusions in this work are reliable and repeatable.
